# The influence of hospital accreditation on nurses’ perceptions of patient safety culture

**DOI:** 10.1186/s12960-024-00920-1

**Published:** 2024-05-28

**Authors:** Islam Ali Oweidat, Huda Atiyeh, Mohammed Alosta, Khalid Al-Mugheed, Amany Anwar Saeed Alabdullah, Majdi M. Alzoubi, Sally Mohammed Farghaly Abdelaliem

**Affiliations:** 1 Faculty of Nursing, Zarqa University, P.O. Box 132222, Zarqa, 13132 Jordan; 2 Riyadh Elm University, Nursing College, Riyadh, Saudi Arabia; 3 Department of Community Health Nursing, College of Nursing, Princess Nourah Bint Abdulrahman University, P.O. Box 84428, 11671 Riyadh, Saudi Arabia; 4 Faculty of Nursing, Al-Zaytoonah University of Jordan, Amman, Jordan; 5 Department of Nursing Management and Education, College of Nursing, Princess Nourah Bint Abdulrahman University, P.O. Box 84428, 11671 Riyadh, Saudi Arabia

**Keywords:** Accreditation, Patient safety, Safety culture, Nurse, Clinical governance

## Abstract

**Objectives:**

Hospitals’ accreditation process is carried out to enhance the quality of hospitals’ care and patient safety practices as well. The current study aimed to investigate the influence of hospitals’ accreditation on patient safety culture as perceived by Jordanian hospitals among nurses.

**Methods:**

A descriptive cross-sectional correlational survey was used for the current study, where the data were obtained from 395 nurses by convenient sampling technique who were working in 3 accredited hospitals with 254 nurses, and 3 non-accredited hospitals with 141 nurses, with a response rate of 89%.

**Results:**

The overall patient safety culture was (71.9%). Moreover, the results of the current study revealed that there were no statistically significant differences between the perceptions of nurses in accredited and non-accredited hospitals in terms of perceptions of patient safety culture.

**Conclusion:**

The current study will add new knowledge about nurses’ perceptions of patient safety culture in both accredited and non-accredited hospitals in Jordan which in turn will provide valid evidence to healthcare stakeholders if the accreditation status positively affects the nurses’ perceptions of patient safety culture or not. Continuous evaluation of the accreditation application needs to be carried out to improve healthcare services as well as quality and patient safety.

## Introduction

Patient safety culture is considered an essential principle and crucial priority for healthcare institutions [1]. The Institute of Medicine (IOM) in 1999, “To Err is Human: Building a Safer Health System” highlighted the need for creating a safety culture in healthcare facilities to manage preventable medical errors [2]. According to the concept of “First, do no harm,” a concept embedded in basic ethical principles and human rights, the primary focus in the delivery of healthcare is to keep patients’ safety [3].

According to WHO, most of patients’ harms or injuries come from surgical procedure, medical errors, hospital-acquired infection, pressure ulcer, and patient fall [4]. Around 50% of these harms and injuries can be prevented while the patient receives care in healthcare facilities [5]. In the UK, the incidence of patients’ harm is reported around 35 incidents every second [6]. In the United States, medical errors become the third cause of death with incidences were 6.3 million patients [5, 7]. In the low- and middle-income countries are not better than developed countries. In Eastern Mediterranean countries, the annual events of patients’ harms or injuries are around 4.4 million and associated with high lifelong disability and rate of death [8]. In Jordan, the annual report of harm or injuries is estimated around 28% [9]. In Palestinian hospitals, found that 59.3% of medical errors were preventable [10]. The consequences of these harms and injuries increase length of stay, increase cost for patient care (around 6 to 29 billion per year), and loss of trust in the delivered healthcare service [6]. For this reason, WHO provided objectives to improve patient safety culture in worldwide healthcare facilities. These objectives are mainly designed to support collaboration among the healthcare teams, improve leadership and working environment to ensure non-punitive response, enhance adequate reporting of any error, and develop policies to enhance the patient safety culture in the healthcare facilities [11].

The Institute of Medicine stressed the need to create a safety culture in all health services [2]. Patient safety culture is a complex principle that needs interdisciplinary teamwork rather than personal performance to improve the quality of care [12]. It depends on the individual and group values, attitudes, and perceptions of providing safe care for their patients [13, 14]. Thus, assessing the perceptions of healthcare providers about patient safety culture can guide the administration of healthcare facilities and the policymakers toward building patient safety culture in their healthcare facilities [15, 16].

Hospital accreditation is a thorough process & management concept and method that is carried out for various healthcare institutions to improve the quality of hospitals’ services as well as patient safety aspects [17]. Moreover, the accreditation process is considered a key component and prioritizes patient safety aspects and the quality of rendered services as well [18]. According to the World Health Organization (WHO), this concept could be the single most substantial process to boost healthcare institutions’ quality [19]. It has been confirmed as the driving process for development and enhancement and includes all of the service delivery components such as processes, structures, and outcomes [20]. The accredited hospitals showed significant improvements in performance compared with non-accredited hospitals, the improvements were shown in creating patient safety systems, disaster planning, staff development and training, and reducing incidence reports [21].

Among the searched literature, there were many research studies that support the fact that the accreditation status of the hospital positively affects the perceptions of nurses toward patient safety culture. For example, a study conducted in Turkey among nurses that revealed hospital accreditation has a positive impact on quality outcomes, especially on the quality of care provided to patients and patient safety perceptions as well [22]. A cross-sectional study found that the nursing accreditation level was positive with patient safety indicators [23]. In a recent study conducted in South Korea, the nurses showed high significance in terms of safety climate with accreditation [24]. Shaw et al. found evidence for positive effects between accreditation, and patient safety [25]. Although numerous hospitals endeavor to obtain the accreditation, several factors affect progress and contribute to reducing healthcare performance-related patient safety, such as staff experience, adverse events, workload, and hospital budgets [26].

Among the searched literature in Jordan, in a recent study, the average positive response rate to the 6 domains of safety culture ranged from 58.54 to 75.63% [27]. In a cross-sectional, descriptive design study among 658 Jordanian nurses, the results provide insight into how nurses perceive patient safety culture [4]. A self-reported questionnaire about patient safety was distributed to the Jordanian nurses, the results obtained that 59% of participants reported good knowledge about patient safety [28]. Up to the researchers’ knowledge; limited literature especially in Jordan was found addressing the influence of hospitals' accreditation on nurses’ perceptions of patient safety culture. Therefore, understanding the influence of accreditation on Jordanian’s nurses’ perceptions of patient safety culture and its dimensions may lead to maintaining the commitment, support, and compliance of healthcare workers and even nurses towards quality principles and patient safety issues inside their hospitals. The study’s primary aim was to investigate patient safety culture among nurses. The secondary aim was to explore associations between patient safety culture and hospital accreditation.

## Methods

### Research design

A descriptive correlational cross-sectional design was used in this study.

### Setting

The study was conducted in different hospitals in Jordan. The target population of this study is nurses working in hospitals with different departments. According to Cohen J [29], the sample size was based on conventional power analysis of medium effect size, a power of 0.80, and a level of significance at 0.05; the estimated sample size was 164 participants for each group (accredited hospitals, and non-accredited hospitals). The hospitals were selected via a simple random sampling technique from accredited hospitals and non-accredited hospitals in Jordan. Hospitals with a capacity of fewer than 60 beds were excluded because of the small number of registered nurses on their duty schedules, and they tended to be peripheral hospitals as well. Convenience sampling enrolls the availability and agreed of nurses to participate in this study with a total of 450 nurses. The final sample was 395 registered nurses who completed the study, in 3 accredited hospitals with 254 nurses, and 3 non-accredited hospitals with 141 nurses, with a response rate of 89%.

The inclusion criteria were nurses who provide direct patient care, have a minimum experience of two years in the hospital where the study is conducted, and have a minimum educational level of bachelor’s degree. The nurses who handle administrative, and managerial roles or clerical work and trainees were exulted from the study.

### Instruments

The hospital survey on patient safety culture (HSPSC) was used to assess the perception of patient safety culture among healthcare providers. The instrument was included in two parts; the first part was demographic and background data such as age, gender, working experience, and accreditation status of the hospital. The second part was an HSPSC instrument composed of three main domains, with 12 sub-domains with a total of 42 items. The first main domain was dimensions of patient safety culture relating to the work area or unit and included seven sub-domains namely teamwork within hospital units (4 items); supervisor/manager expectations and promotion of safety (4 items); organizational action for learning and continuous improvement (3 items); feedback and communication in relation to error (3 items); communication openness (3 items); staffing (4 items); non-punitive response towards error (3 items).

The second main domain was dimensions that explore aspects of safety culture in a hospital and included three sub-domains namely management support about patient safety (3 items); teamwork across units (4 items); hands-off and transitions (3 items).

The third main domain was dimensions of outcome measurements and included two sub-domains was overall perceptions of safety (4 items); frequency of reporting events (3 items). In this study, the Cronbach’s *α*, was 0.84.

### Data collection

The researcher started data collection between April to June 2021, by sending a link in Google Form and sharing it between the nurses, which included the aim of the study and contact information. By clicking on the “Agree and Proceed” button it was considered agreed to participate. Also, participants were informed that data would be kept at a high level of confidentiality and would not affect their job.

### Data analysis

Data analysis was done using the Statistical Package for the Social Sciences version 23 (SPSS 23). Descriptive analysis was utilized to describe the demographic characteristics of the participants. Scores from the HSPSC were obtained to describe the status of patient safety culture in hospitals. Each item is rated by a five-point Likert scale with scores ranging from 1 = strongly disagree to 5 = strongly agree. Independent sample *t* test was used to detect if there is a statistically significant difference between accredited and non-accredited hospitals toward patient safety culture.

## Results

Table 1 shows the demographics of participants with mean age were (29.1 ± 6.7). The majority of participants were female in both groups accredited (82.1%, non-accredited 86.7%). Half of the participants had experiences of more than 11 years (66.1%) in non-accredited. Most participants work in accredited hospitals (67.4%).

**Table 1:** Demographics of participants

	Accredited		Non-accredited	
	*N*	%	*N*	%
Age				
Mean 29.1 ± 6.7				
20–25 years	20	8.3	8	1.4
26–30 years	77	43.5	81	47.1
> 31 years	115	48.2	94	51.5
Gender				
Male	54	17.9	44	13.3
Female	143	82.1	154	86.7
Years of experience				
1–5 years	53	7.4	35	16.7
6–10 years	105	45.1	39	17.2
> 11 years	118	47.5	75	66.1
Accreditation hospital status				
Accredited	254	67.4	141	32.6

In regard to the dimensions of patient safety culture, the overall patient safety culture was (71.9%). The highest positive response of patient safety was teamwork within hospital units (83.3%), hands-off and transitions (81.5%), Communication openness (79.1%), and feedback and communication in relation to error (77.5%). The lowest positive response was supervisor/manager expectations and promotion of safety (58.1%), followed by frequency of reporting events (59.7%) Table 2.

**Table 2:** Perceptions of participants related to patient safety culture

	Cronbach’s *α*	Accredited positive response %	Non-Accredited positive response %	Average percentage of positive response % in both groups
Teamwork within hospital units	0.78	85.1	82.5	83.3
Supervisor/manager expectations and promotion of safety	0.58	54.7	60.6	58.1
Organizational action	0.57	71.4	68.2	70.1
Feedback and communication in relation to error	0.79	79.5	75.7	77.5
Communication openness	0.48	74.7	81.2	79.1
Staffing	0.51	71.5	62.9	66.8
Non-Punitive response towards error	0.67	75.7	66.4	71.0
Management support for patient safety	0.70	69.8	81.9	76.3
teamwork across units	0.66	74.7	70.5	71.7
hands-off and transitions	0.77	89.7	77.4	81.5
Overall perceptions of safety	0.59	51.7	72.9	68.4
frequency of reporting events	0.75	62.8	56.8	59.7

In regard to the other research question which was about whether there is a statistically significant difference between accredited and non-accredited hospitals in terms of perceptions of patient safety culture; the results of the current study revealed that there were statistically significant differences between the perceptions in accredited and non-accredited hospitals in terms of patient safety culture Table 3.

**Table 3:** Comparison of total patient safety culture and accredited and non-accredited hospitals

	Total patient safety culture	*p*
Accredited hospitals	50.3 ± 2.1	0.03
Non-accredited hospitals	49.2 ± 1.5	0.11

The study found no significant predictors for accredited hospitals and non-accredited hospitals based on participants’ demographics. Nurses who were working in accredited hospitals showed more positive related to patient safety culture than nurses who were working in non-accredited hospitals in terms of gender and years of experience Table 4.

**Table 4:** Multiple linear regression analysis results: factors associated with accredited hospitals and non-accredited hospitals

	Accredited hospitals			Non-accredited hospitals		
	*B*	Std. Error	*p*	*B*	Std. Error	*p*
Age	0.154	0.125	0.211	0.592	0.314	0.577
Gender	0.318	1.40	0.236	0.704	0.205	0.846
Years of experience	0.208	1.54	0.224	− 0.271	.089	0.954

*p* < 0.05

## Discussion

Patient safety and accreditation concepts are global concerns, so, further research studies should be conducted to explore the reasons behind the accreditation process does not guarantee patient safety culture and reporting culture [26]. A positive patient safety culture is crucial for every healthcare institution in Jordan and even in the world to provide safe care and to avoid any harm to patients.

Up to the best knowledge of the researchers; this is the first research study that investigates the influence of accreditation on the perceptions of patient safety culture among nurses. Actually, the results of the current study showed that the highest composite frequency of patient safety reflected nurses’ positive perception of teamwork within hospital units, while the lowest composite reflected positive opinion were the frequency of reporting errors. These results are considered consistent with the results of Khater and colleagues which they found that the highest dimension that was perceived positively by nurses in Jordan was “team work within units” [4]. Additionally, the results of the current study are considered consistent with the results of Al-Mugheed and colleagues which they found that the highest domain that was perceived positively by the surveyed participants was teamwork within units [12]. On the other hand, the current results are inconsistent with the results of Rao and colleagues [30]. In other words, the participants of the current study viewed their environment as supportive of each other; there is mutual respect between staff.

One of the alarming results of the current study was related to the frequency of reporting errors among Jordanian nurses which was the lowest dimension in terms of perceptions of patient safety culture; it may be due to the complexity of the healthcare system in the world and in Jordanian context (high technical equipment and inadequate communication; continuous evolutions in healthcare); patients are more prone to be harmed or injured while receiving care in the healthcare facilities [31, 32]. Some healthcare providers also feared that their mistakes would have been held against them, Patient safety issues could be improved in Jordanian hospitals if JUST culture was fostered, which means recognizing the errors as flaws in the system, rather than a single individual’s failure, as well as they could be learned lessons [33]. There is a need to have an adequate understanding about patient safety culture to be implemented in healthcare facilities [34]. This can be achieved by assessing the current patient safety culture in the healthcare facilities [35, 36].

The results of the current study revealed that there is no statistically significant difference was found between healthcare providers in accredited versus non-accredited hospitals in terms of perceptions toward patient safety culture. These results were considered consistent with the results of Shaikh study, in which he had investigated the impact of accreditation on safety practices perspectives of healthcare workers in India and it was revealed that there is a significant increase in the number of incidents reporting practices after the accreditation process of the hospitals [19]. In fact, the researcher does believe that this issue is debatable since some research studies support the idea that the accreditation status of any healthcare institution is highly correlated with safety culture perceptions [37, 38], however, other research studies revealed that there is no significant correlation between accreditation status of the healthcare setting and incidents reporting practices. For example, the study that was conducted in the Jordanian context (2015) by AbuAlRub and colleagues revealed that healthcare providers in accredited and non-accredited hospitals did not differ statistically either in their incident reporting practices or their awareness of the reporting system [39]. However, nursing managers in accredited hospitals are required to maintain patient safety culture aspects such as incident reporting and communication among health workers after the accreditation process which needs actual commitment and support from all staff in hospitals.

### Limitations of the study

Although the current study has many strength points; the results of the current study were subjected to several limitations. One limitation of this study resulted from its convenience sampling method; a method based on the selection of participants who were accessible electronically at the time of data collection. However, many nurses from different settings were selected to expand the generalizability of findings, also the represented sample was conducted in one country. Additionally, the current results revealed the perspectives of healthcare workers only; but actually, there is a need to assess the patient safety culture from the perspectives of clients and recipients of care being rendered.

## Conclusion

To enhance the perceptions of patient safety culture among nurses, they need to be sensitized regarding patient safety issues, especially on standard treatment protocols, incident reporting practices, and communication mechanisms through continuing professional development (CPDs) or job training. The current study will add new knowledge about nurses’ perceptions of patient safety culture in both accredited and non-accredited hospitals in Jordan which in turn will provide valid evidence to healthcare stakeholders if the accreditation status positively affects the nurses’ perceptions toward patient safety culture or not. As well as it clearly shows the areas of strength and areas that are in need of improvement for nurses to expand their practice toward patient safety culture.

## Data Availability

The data that support the findings of this study are available on request from the corresponding author.
